# Endogenous miRNA Sponge LincRNA-ROR promotes proliferation, invasion and stem cell-like phenotype of pancreatic cancer cells

**DOI:** 10.1038/cddiscovery.2017.4

**Published:** 2017-05-29

**Authors:** Zhiqiang Fu, Guolin Li, Zhihua Li, Yingxue Wang, Yue Zhao, Shangyou Zheng, Huilin Ye, Yuming Luo, Xiaohui Zhao, Lusheng Wei, Yimin Liu, Qing Lin, Quanbo Zhou, Rufu Chen

**Affiliations:** 1 Department of Hepato-Pancreato-Billiary Surgery, Sun Yat-sen Memorial Hospital, Sun Yat-sen University, Guangzhou, China; 2 Guangdong Provincial Key Laboratory of Malignant Tumor Epigenetics and Gene Regulation, Medical Research Center, Sun Yat-sen Memorial Hospital, Sun Yat-sen University, Guangzhou, China; 3 Department of Medical Oncology, Sun Yat-sen Memorial Hospital, Sun Yat-sen University, Guangzhou, China; 4 Department of Endocrinology, The First Affiliated Hospital, Jinan University, Guangzhou, China; 5 Department of Gastroenterology, The First Affiliated Hospital, Sun Yat-sen University, Guangzhou, China; 6 Department of Radiotherapy, Sun Yat-sen Memorial Hospital, Sun Yat-sen University, Guangzhou, China

## Abstract

The long intergenic non-coding RNA, regulator of reprogramming (linc-ROR) is an oncogene and plays a key role in the embryonic stem cell maintenance and is involved in cancer progression. The objective of this study was to analyze linc-ROR expression in pancreatic ductal adenocarcinoma (PDAC) and determine the regulation effects of linc-ROR on proliferation and invasion of cancer cells, as well as properties of cancer stem-like cells (CSLCs). In this study, we found that linc-ROR was up-regulated in PDAC tissues and related to poor prognosis. Linc-ROR knockdown in pancreatic cancer cells inhibited cell growth and arrested in G1 phrase. Suppressed linc-ROR expression also attenuated cancer cell migration, invasion, and epithelial-mesenchymal transition. We observed that linc-ROR expression was increased in CSLCs. Importantly, linc-ROR knockdown impaired the properties and tumorigenesis of pancreatic CSLCs *in vivo*. Mechanistically, we found that linc-ROR functioned as a competing endogenous RNA (ceRNA) to several tumor suppressor microRNAs, particularly some members of let-7 family. We conclude that, as a crucial oncogene, linc-ROR promotes cell proliferation, invasiveness and contributes to stem cell properties of CSLCs in PDAC via acting as a ceRNA to regulate function of microRNAs. The linc-ROR is a potential therapeutic target for PDAC.

## Introduction

Pancreatic ductal adenocarcinoma (PDAC) holds one of the most malignant types of human cancer. Despite tremendous efforts, PDAC is still related to a short survival with about 7 percent five-year survival rate now. Obviously, it is important to further understand the mechanism of PDAC development and dig innovative therapy approaches. In recent years, it is well acknowledged that non-coding RNAs (ncRNAs) play a vital role in both normal physiology and diseases.^1^ Moreover, long non-coding RNAs (lncRNAs), including antisense lncRNA, Intronic transcript, large intergenic noncoding RNA (lincRNA), promoter-associated lncRNA and UTR associated lncRNA, defined as ncRNA >200 nucleotides in length, are attracting tremendous attention as its important role in regulating vital cellular functions.^1^ So far, a large range of function of lncRNAs has been identified, such as regulation of apoptosis and invasion,^2^ remolding of induced pluripotent stem cells (iPS),^3^ management of tissue differentiation,^4^ and grasp of cell fate.^5^ Importantly, many lncRNAs have been identified as being cancer-specific,^6,7^ these lncRNAs might be employed as novel biomarkers or therapeutic targets.

Besides microRNAs, a significant portion of the non-coding RNAs, including long non-coding RNAs and pseudogenes, harbors miRNA-response elements (MRE).^8^ Recent studies described a novel interplay among non-coding RNAs harboring MRE. These MRE-harboring non-coding RNA transcripts act as competing endogenous RNAs (ceRNAs) that compete for a common pool of miRNAs and regulate the expression of protein-coding RNAs.^9^ In previous reports, a muscle-specific LncRNA MD1 has been reported to control muscle differentiation by functioning as a ceRNA targeting miR-133.^4^ In addition, the non-coding 3′UTR of HMGA2 transcript can disturb let-7 activity by changing miRNA targeting genes in lung cancer.^10^ In addition, lncRNA HULC, as a ceRNA, down-regulates a series of miRNAs, including miR-372 and thus promotes liver cancer development.^11^ The ceRNA mechanism links lncRNAs and miRNAs in the post-transcriptional network of tumor pathogenesis, which may explain disease processes and present opportunities for new therapies. However, its role in pancreatic cancer remains unknown.

Linc-ROR was first discovered as a sponsor of remolding of human iPS, where it was previously hypothesized to promote the transcription of core pluripotency factors including Oct4, Sox2 and Nanog based on the chromosome-modifying functions of many other reported lincRNAs.^3,12^ But recent studies showed that linc-ROR actually functions as a microRNA sponge for miR-145 to increase the expression of mir-145 targets, such as OCT4, SOX2 and Nanog.^12,13^ Moreover, linc-ROR has been demonstrated to make an important impact in the regulation of hypoxia signaling pathways in liver original cancer cells.^14^ In breast cancer, Hou *et al.* reported that lincRNA-ROR could induce epithelial-to-mesenchymal transition (EMT) and promote carcinogenesis and development of breast cancer by targeting miR-205.^15^ As many embryonic stem cells-related genes often play a similar function in cancer stem cells (CSCs),^16,17^ it is valid to hypothesize that linc-ROR may also implement a role in regulating CSCs properties. In addition, the fact that linc-ROR can act as a sponge not only for miR-145 but also for let-7,^15^ miR-205^15^ and other potential miRNAs,^12^ and findings that other lncRNAs with ceRNA activity usually have several target miRNAs,^9^ attracted us to speculate that linc-ROR may also affect CSCs properties through regulating one or more important tumor-suppressor miRs.

In this study, we aimed to explore the role of linc-ROR in regulation of proliferation, invasion and the CSC properties of cancer stem-like cells (CSLCs) in pancreatic cancer. We showed that linc-ROR knock-down impaired the proliferation, colony formation, migration, invasion ability and decreased the expression of EMT-related genes. Moreover, we identified an important role of linc-ROR in the maintaining of CSC properties of CSLCs in pancreatic cancer cells. Furthermore, we found that let-7, miR-7 and miR-451, which have been known to have key roles in repressing tumor proliferation, invasion and CSLCs properties, were ceRNA targets of linc-ROR. We thus identify an important and novel regulatory mechanism of linc-ROR in pancreatic cancer progression.

## Results

### Linc-ROR is over-expressed in human PDAC tissues and related to poor prognosis

**Figure 1:**
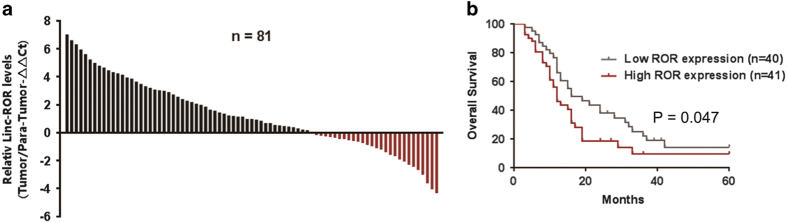
Linc-ROR is over-expressed in human PDAC tissues and is related to poor prognosis. (**a**) Relative expression levels of linc-ROR in 81 PDAC tissues compared with adjacent para-tumor tissues were evaluated by qRT-PCR. Results are shown as ^−ΔΔ^CT values. (**b**) The patients were divided into two groups based on linc-ROR levels. The log-rank test (two-sided) was used to compare differences between groups. The Kaplan–Meier curves show analyses of overall survival.

To investigate the role of linc-ROR in pancreatic cancer, we first evaluated the linc-ROR expression in paired tumor and para-tumor tissues from 81 clinical PDAC specimens. Quantitative realtime-PCR(qRT-PCR) analysis demonstrated that linc-ROR expression was up-regulated in tumor tissues compared with para-tumor tissues (Figure 1a). In addition, log-rank analysis indicated that overall survival was significantly reduced in patients with higher linc-ROR expression (*P*=0.047) (Figure 1b). These results indicated that increased level of linc-ROR may be positively correlated with the progression of pancreatic cancer.

### Linc-ROR regulates pancreatic cancer cell proliferation

**Figure 2:**
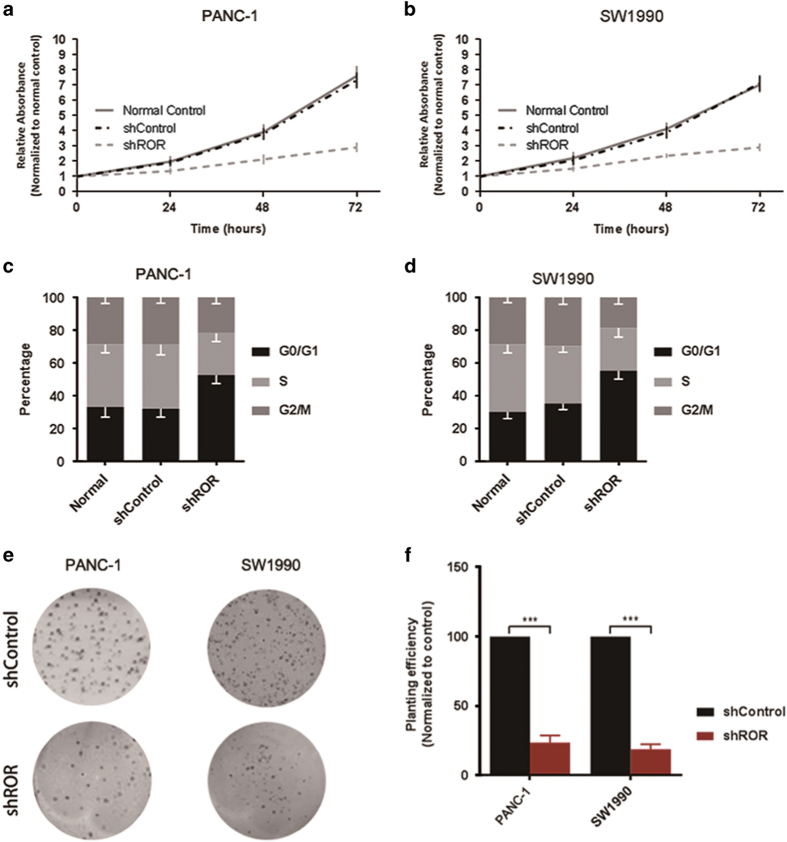
Linc-ROR regulates pancreatic cancer cell proliferation. (**a**) Results of MTT assays showing effect of linc-ROR knockdown on cell proliferation in PANC-1 cells. (**b**) Results of MTT assays showing effect of linc-ROR knockdown on cell proliferation in SW1990 cells. (**c**) Cell cycle analysis of PANC-1 cells and (**d**) SW1990 cells stably transfected with shRNA-mediated scramble (shControl) or shRNA-mediated silencing of linc-ROR (shROR). (**e**) Representative images of clone formation assays performed in PANC-1 cells and SW1990 cells transfected with shControl or shROR. (**f**) Colony formation was analyzed, the number of colonies of shROR group were normalized to that of shControl group. ErROR bars represent the mean±S.D. of triplicate experiments. Statistical significance was calculated using the Student's *t* test or ANOVA tests. ***P<0.001.

Stable linc-ROR knock-down cell lines (PANC-1 and SW1990) were established by using retrovirus infection, and the silencing effect was confirmed by qRT-PCR. To assess the role of linc-ROR in pancreatic cancer cell proliferation, we performed MTT assays on control and linc-ROR-suppressed cells. The depletion of linc-ROR expression reduced cell proliferation significantly compared with shControl cells both in PANC-1 cell line (Figure 2a) and SW1990 cell line (Figure 2b). We further analyzed the effect of linc-ROR on cell cycle, and found linc-ROR suppression obviously reduces number of cells in the S-phase and increase in the G0/G1 phase (Figures 2c and d). As expected, Knockdown of linc-ROR significantly decreased the clone formation of both cells (Figures 2e and f).

### Linc-ROR regulates pancreatic cancer cell migration, invasion and EMT

**Figure 3:**
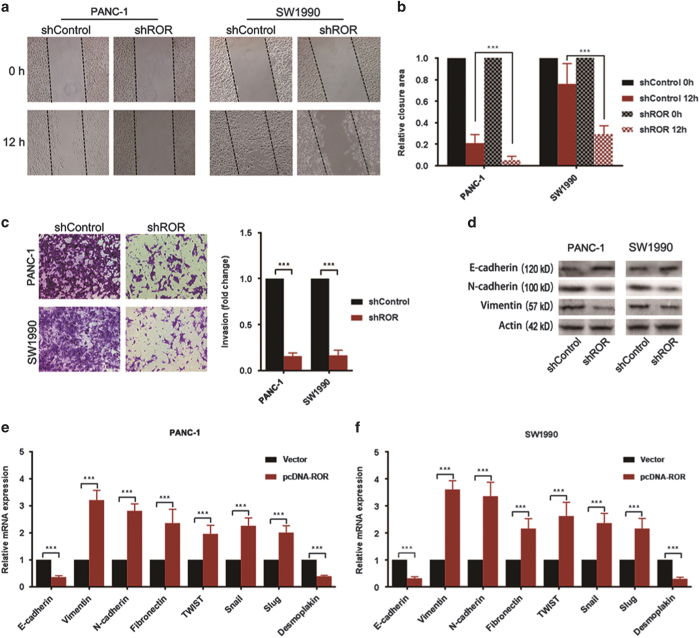
Linc-ROR regulates pancreatic cancer cell migration, invasion and EMT. (**a**) The scratch wound healing assay was performed in PANC-1 cells and SW1990 cells to assess the effect of linc-ROR on cell mobility in the presence of Mitomycin C. Dashed lines marked boundaries of the initial scratch. (**b**) Bars represented the percentage of wound healing, the area between dashed lines of shControls were set as 1. (**c**) Invasion assay were performed to assess the effect of linc-ROR on cell invasive ability of PANC-1 cells and SW1990 cells. Photos were representative fields of invasive cells on the membrane. (**d**) Western blot analysis of E-cadherin, N-cadherin and Vimentin was performed in PANC-1 cells and SW1990 cells stably transfected with shRNA-mediated scramble (shControl) or shRNA-mediated silencing of linc-ROR (shROR). (**e**) PANC-1 cells and (**f**) SW1990 cells were stably transfected with pcDNA3 empty vectors (NC) or pcDNA3 ROR overexpressing vectors (ROR), and the relative expression levels of mRNA expression of EMT-related genes were determined using qRT-PCR. Results are shown as ^2ΔΔ^CT. ErROR bars represent the mean±S.D. of triplicate experiments. Statistical significance was calculated using the Student's *t* test or ANOVA tests. ***P<0.001.

We evaluated the effects of linc-ROR on cell migration and invasion of PANC-1 with relatively moderate level of linc-ROR expression, and on SW1990 cells which expresses relatively higher levels of linc-ROR. We first examined the effect of linc-ROR knockdown on PANC-1 and SW1990 cells migration using wound healing assay in the presence of Mitomycin, and found that cells stable transfected with linc-ROR shRNA had significant slower motility (relative wound closure proportion) compared with cells stable transfected with scramble RNA (Figures 3a and b). Furthermore, we investigated whether suppression of linc-ROR expression would impair cell invasion ability. Matrigel invasion assay showed that stable knockdown of linc-ROR dramatically reduced the invasion of PANC-1 and SW1990 cells (Figure 3c). We next examined whether the silencing of linc-ROR expression can suppress the EMT process. Cells were seeded 1×10^6^ per well on 6-well plate, and were incubated with medium containing 10 ng/ml transforming growth factor-beta (TGF-*β*) and 50 ng/ml stromal-derived factor (SDF-1) for 72 h. Western blot analyses showed that stable knock-down of linc-ROR was associated with impaired down-regulation of epithelial marker E-cadherin and up-regulation of mesenchymal markers N-cadherin and Vimentin (Figure 3d). Furthermore, we examined the effect of up-regulating linc-ROR expression on other EMT-related genes by qRT-PCR, and results in contrast with the above findings are revealed (Figures 3e and f).

### Linc-ROR is over-expressed in pancreatic CSLCs

**Figure 4:**
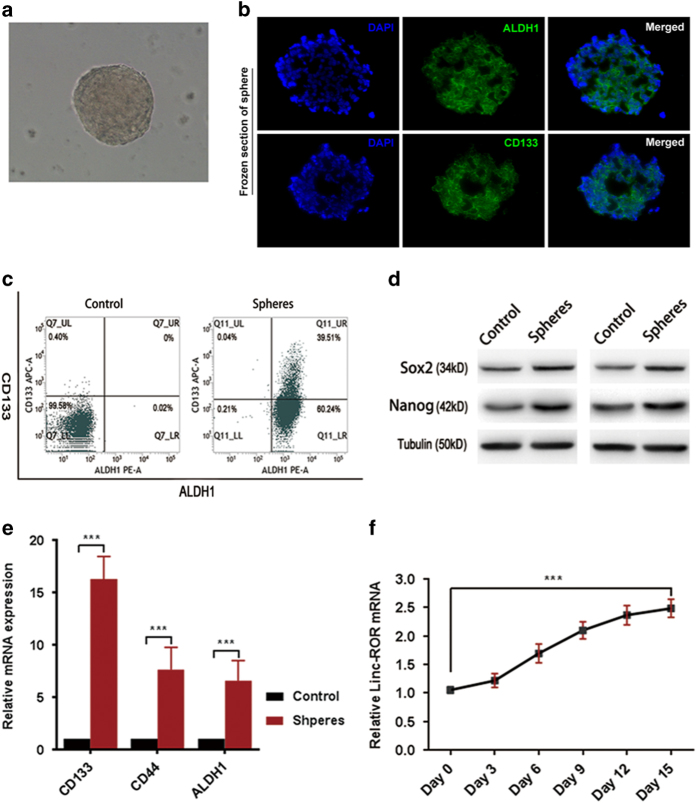
Linc-ROR is over-expressed in pancreatic CSLCs. (**a**) Representative light microscope images of spheres in PANC-1 cells. (**b**) Immunofluorescence staining and confocal imaging for CD133 and ALDH1 in PANC-1 spheres. (**c**) Flow cytometry analysis for CD133 and ALDH1 PANC-1 adherent cells (control) and spheres. (**d**) Western blot analysis of SOX2 and Nanog in PANC-1 spheres compared with adherent cells as control. (**e**) Expression of CD133, CD44 and ALDH1 in PANC-1 spheres relative to adherent cells (controls) were determined using qRT-PCR. Results are shown as 2ΔΔCT. (**f**) qRT-PCR showing the change of linc-ROR expression levels along with the formation of shperes. Results are shown as ^2ΔΔ^CT. ErROR bars represent the mean±S.D. of triplicate experiments. Statistical significance was calculated using the Student's *t* test or ANOVA tests. ***P<0.001.

We obtained CSLCs from PANC-1 cells, as described in Materials and methods. The representative images of ×100 light microscope view of spheres are shown in Figure 4a. As the sphere-forming process is intended to enrich the potential CSLC subpopulations, we further detected the CSCs markers in spheres. Spheres were analyzed for the expression of previously reported CSLC-related markers and genes including CD44, CD133, ALDH1, SOX2 and Nanog. We found that the above markers are highly enriched in cells of spheres (Figures 4d and e). In addition, the proportion of CD133 and ALDH1 double positive cells were dramatically increased in cells of spheres (Figures 4b and c). These data indicate that the CSLCs were enriched from PANC-1 cells through sphere formation. As linc-ROR was reported high-expressed in embryonic stem cells and pluripotent stem cells, we further examined the change of linc-ROR expression levels following sphere formation. As expected, the expression of linc-ROR was increased 2.482 fold in spheres compared with control normal PANC-1 cells.

### Linc-ROR regulates the CSC properties of stem cell-like pancreatic cancer cells

**Figure 5:**
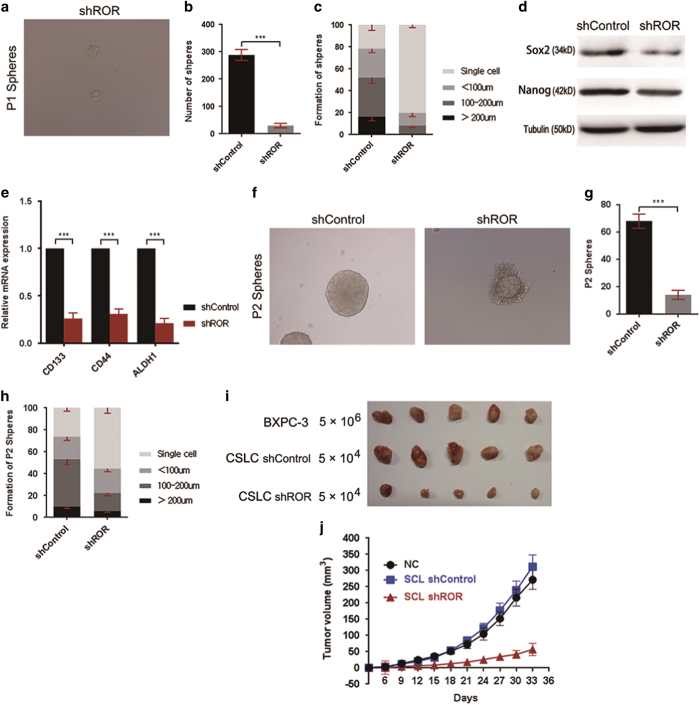
Linc-ROR regulates the CSC properties of stem cell-like pancreatic cancer cells. (**a**) Representative light microscope images showing spheres derived from PANC-1 cells stably transfected with shRNA-mediated silencing of linc-ROR (shROR). (**b**) Number of spheres derived from shROR PANC-1 cells compared with shControl PANC-1 cells. (**c**) Distribution proportion of spheres based on size in shROR PANC-1 cells compared with shControl cells. (**d**) Western blot analysis of SOX2 and Nanog in shControl PANC-1 sphere cells compared with shROR PANC-1 sphere cells. (**e**) Expression of CD133, CD44 and ALDH1 in shControl PANC-1 sphere cells relative to shROR PANC-1 sphere cells. (**f**) Representative light microscope images of P2 spheres derived from shControl and shROR cells of P1 spheres. (**g**) Comparison of number of P2 spheres derived from P1 spheres. (**h**) Comparison of size distribution of P2 spheres derived from P1 spheres. (**i**) The effect of linc-ROR on sphere tumorigenicity was investigated *in vivo* through injecting the indicated number of PANC-1 cells stable transfected with scramble RNA, CSLC shControl (cells of P1 shperes derived from PANC-1 cells stably transfected with vectors containing scramble RNA), and CSLC shROR (cells of P1 shperes stably derived from PANC-1 cells stably transfected with vectors expression shROR) cells. (**j**) Tumor volume was determined as described in Materials and methods section. ErROR bars represent the mean±S.D. of triplicate experiments. Statistical significance was calculated using the Student's *t* test or ANOVA tests. ***P<0.001.

Given the important role of linc-ROR in maintaining stem cell properties, we next evaluated the effect of linc-ROR in regulating the stem cell-like properties of CSLCs derived in the above part. First, we compared the sphere formation ability between PANC-1 cells stably transfected with scramble RNA (shControl) and linc-ROR knock-down cells (shROR). We found that linc-ROR knock-down dramatically impaired the sphere formation of PANC-1 cells. The typical morphological feature of spheres derived from linc-ROR knock-down cells is shown in Figure 5a. The comparisons of total number of spheres derived from a same number of initial PANC-1 cells are shown in Figure 5b, a 10-fold reduction in formation number were observed in shROR cells. Also, the diameter of spheres derived from shROR cells was significantly decreased (Figure 5c). In addition, we picked spheres from both group, and examined the CSC markers in these cells by western blot and qRT-PCR, which demonstrated a decreased CSC markers in spheres derived from shROR cells (Figures 5d and e). Furthermore, we selected spheres of 100–200 *μ*m in diameter from both group, and performed the secondary passage sphere formation test. After incubation in the sphere-culture medium about 10 days, the secondary passage spheres formed well by P1 cells of control group. In contrast, P1 cells with suppressed linc-ROR expression demonstrated impaired P2 sphere formation ability both in morphological features (Figure 5f), number (Figure 5g) and size (Figure 5h). At last, we evaluate the effect of linc-ROR on pancreatic CSLCs tumorigenicity. We analyzed the *in vivo* tumorigenicity of PANC-1 cells, cells of P1 spheres in nude mice (Figures 5i and j). As expected, injection of 1×10^4^ cells of P1 spheres derived from PANC-1 cells stable transfected with scramble shControl RNA showed comparable tumorigenicity compared injection of 1×10^6^ PANC-1 cells stable transfected with scramble shControl RNA. In addition, as expected, P1 spheres derived from shROR PANC-1 cells demonstrated a much weaker tumorigenicity.

### Linc-ROR functions as an endogenous microRNA Sponge in pancreatic cancer cells

**Figure 6:**
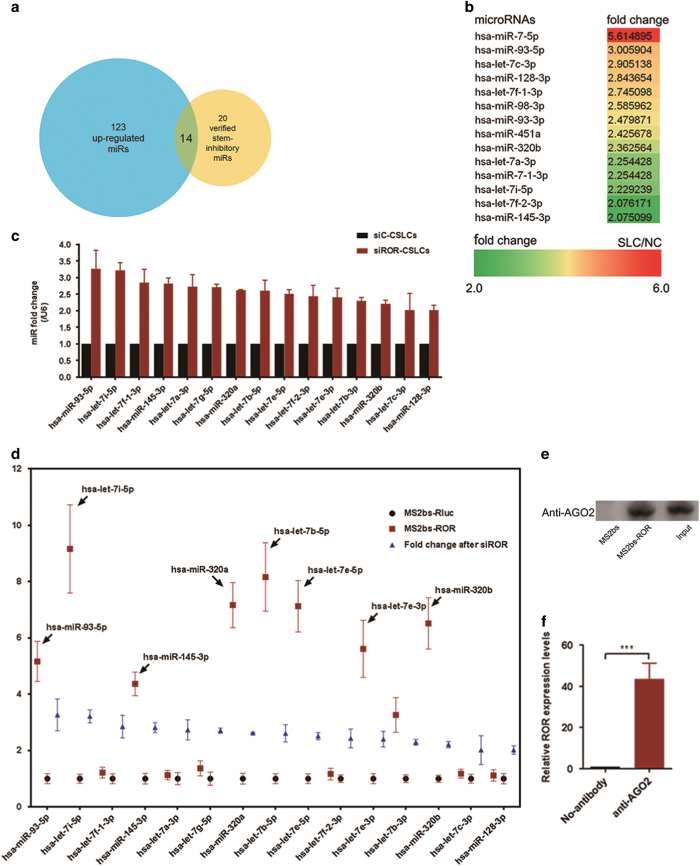
Linc-ROR Functions as an Endogenous microRNA Sponge in pancreatic cancer cells. (**a**) Total RNAs of PANC-1 cells (NC) and PANC-1 derived stem cell-like cells (SCLCs) were used for a microarray analysis, 123 microRNAs were found decreased more than two-fold in SCLCs compared with PANC-1 cells. By comparing with the confirmed tumor and stemness inhibitory microRNAs, 14 overlaping genes were determined. (**b**) The heatmap of the 14 determined miRs. The numbers indicated the fold change of gene expression (Fold SCLC/NC) determined by microarray. (**c**) The effect of linc-ROR on microRNA expression in PANC-1 cells and SCLCs were evaluated by using qRT-PCR. A total of 15 microRNAs elevated more than two-fold after linc-ROR knock-down were shown. (**d**) The binding ability of the above 15 miRs to linc-ROR full-length transcripts were evaluated by cDNA of linc-ROR combined with MS2-binding sequences (MS2bs) and its binding protein MS2BP-YFP in SCLCs. (**e**) The binding efficiency of linc-ROR to AGO2 protein in SCLCs were determined by RIP assay, (**f**) Amount of linc-ROR binding with AGO2 were evaluated by using qRT-PCR. ErROR bars represent the mean±S.D. of triplicate experiments. Statistical significance was calculated using the Student's *t* test or ANOVA tests. ***P<0.001.

It has been reported that linc-ROR can function as molecular sponges to bind relative miRNAs to affect their function, and down-regulating the expression of targeted-miRs.^12^ Therefore, the down-regulated miRs following sphere formation might be the potential target of linc-ROR. We employed microarray gene expression analysis (miRCURY LNA Array, v.18.0, Exiqon) using PANC-1 cells and spheres derived from PANC-1 cells (Supplementary Table 1). As a result, 123 miRs were found down-regulated more than two times in the cells of spheres. By comparing miRs have been reported to have inhibitory effect on stem properties of cancer cells (Supplementary Table 2), we found fourteen overlapping microRNAs (Figures 6a and b). Notably, the fourteen overlapping genes contain lots of let-7 family members, which intrigued us to explore the effect of linc-ROR on let-7 expression. Besides, we noticed that there is only one different nucleotide sequence between miR-320a and hsa-miR-320b, and microarray showed >1.5 fold change of miR-320a, we also further tried to evaluate the effect of linc-ROR on miR-320a expression. Altogether, a total of twenty-seven miRs (Supplementary Table 3) were included for the next step. We first verified the microarray results through re-analyzing the expression of these 27 genes in PANC-1 cells and spheres by using qRT-PCR. Result of PCR were close to that of microarray (Supplementary Figure 1), and the linear regression analysis revealed a correlation between PCR results and microarray results (Supplementary Figure 2, *P*<0.001), which indicating that the microarray results were reliable.

Next, we suppressed linc-ROR expression in CSLCs through transfection of siRNA. Compared with controls (siC, transfection of non-targeting siRNA), expression levels of almost all these 27 miRs were increased, and there were 15 miRs increased more than two fold (Figure 6c). We employed RNAhybrid to search putative complementary sequences for the seed region of these 15 miRs in linc-ROR, and found there are many potential interaction sites between these miRs and linc-ROR (Supplementary Table 4). To validate the relationship between expression of these miRs and the ceRNA activity of linc-ROR, we further use the RNA immunoprecipitation (RIP) analysis with MS2-binding protein (MS2bp), which specifically binds RNAs that contain MS2-binding sequences (MS2bs), when they are co-expressed. Vectors expressing lin-ROR containing MS2bs elements (ROR-MS2bs) connected behind the sequence were constructed, and we co-transfected ROR-MS2bs, Flag-MS2bp, a pool of miRs including the above 15 miRs into PANC-1 cells. Controls were cells transfected with a same amount of the pool of miRs, MS2bs vector, and Flag-MS2bp. Subsequently, we performed RIP assay using the ANTI-FLAG M2 Affinity Gel. Real-time PCR assays showed that many let-7 family members, including hsa-let-7i-5p, hsa-let-7b-5p, hsa-let-7e-5p, hsa-let-7e-3p, hsa-let-7b-3p, hsa-let-7c-3p, were enriched in ROR-12*MS2bs RNA in contrast to the controls (Figure 6d). miR-93-5p, miR-145-3p, miR-320a, and miR-320b were also found enriched (Figure 6d). Because studies have shown that linc-ROR suppress miR expression through Ago2-related RNA-induced silencing complexes (RISCs), we then confirmed that linc-ROR was recruited to AGO2 (Figures 6e and f), which was in consistent with previous reports.

## Discussion

Thousands of lincRNAs are discovered in mammalian and play various roles in transcriptional regulation, epigenetics and occurrence and development of cancer. So far, lots of lincRNAs are researched through chromatin characteristics analysis and mass sequencing,^18^ functional exploration has just begun. Functional studies have showed that several lincRNAs take part in pathogenesis of human cancers, implement as oncogenes or antioncogenes. In this study, we revealed that linc-ROR is commonly overexpressed in PDAC and its overexpression and poor prognosis have a significantly positive correlation, revealing oncogenic function of linc-ROR. The further functional experimenters found that linc-ROR knockdown decreases pancreatic cancer cell proliferation, ability of colony formation, activity and invasion *in vitro*, and impaired the stem cell-like features of pancreatic cancer cell and tumorigenicity potential *in vitro* and *in vivo*.

LincRNAs have been found to interplay with various proteins, thereby permitting scaffolding functions and combinatorial control.^19^ For example, Xist and Kcnq1ot1 are recognized as interacting with and recruiting histone modification complexes.^20^ LncRNA HOTTIP directly unites WDR5 protein and targets WDR5/MLL complexes across HOXA, promoting HOXA genes transcription.^21^ During DNA damage, Linc-p21 is up-regulated by p53 and binds hnRNPK to control gene expression.^22^ Another well-known lincRNA, HOTAIR, can stimulate cancer metastasis through interacting with Polycomb repressive complex 2 to regulate histone H3 lysine 27 methylation.^23^ However, unlike the above mechanism that LncRNA act as protein recruiter and locator, recent data indicate that ncRNAs can regulate gene expression by competing for miRNA binding termed competing endogenous RNA (ceRNA).^24^ Till now, two lincRNAs, linc-MD1 and linc-ROR have been identified to act as ceRNA and, in doing so, inhibits their target microRNAs-mediated messenger RNA (mRNA) degradation.^4,12^

Linc-ROR was first identified as an endogenous sponge inhibiting the differentiation of ESC by targeting miR-145.^12^ Recent studies revealed a tumor-promoting role of linc-ROR in breast cancer,^15^ endometrial cancer.^25^ Most recently, Takahashi *et al.*^14^ revealed that linc-ROR regulates hypoxia signaling pathways by inhibiting the function of miR-145 in hepatic cancer cells. Importantly, linc-ROR also revealed potential ceRNA activity targeting other miRs exhibits tumor-suppressor activity, such as miR-181a,^13,26,27^ miR-99b,^13,28^ and let-7a-5p.^15,29^ The above findings showed that linc-ROR was a potential carcinogene. In this study, we uncovered a critical role for linc-ROR in pancreatic cancer cell proliferation, showing that linc-ROR knockdown arrests cells at the G1 phase. Besides, our data demonstrated that suppression of linc-ROR dramatically impaired the expression of mesenchyme markers under the treatment of TGF-*β* and SDF-1. Notably, at the microenvironment level, both TGF-*β* and SDF-1 are major key mediators of the dialogue between cancer and stromal infiltrating cells,^30,31^ which are widespread involved in the regulation of cancer cell proliferation, differentiation, invasion, and inflammation.^32^ Therefore, it seems that linc-ROR would continue to play a role in tumor progression under complex tumor microenvironment.

Cancer stem cells (CSCs) are stirring field for cancer studies, and provide a novel target for tumor treatment. Cancer stem cells are defined as rare cells in cancer tissues with indefinite potential for self-renewal that drives tumorigenesis.^33,34^ As the identification of CSCs or cancer stem-like cells (CSLCs) in pancreatic cancer,^35,36^ pancreatic CSCs/CSLCs have emerged as a possible, attractive explanation for the highly incorrigible therapy resistance of pancreatic cancer.^37^ Thus the molecular mechanism contributing to the maintenance of pancreatic CSCs/CSLSc properties have been an emerging focus of recent research. As linc-ROR was shown to play an important part in preserving the pluripotency of human embryo stem cells,^13^ here we studied emphatically on the role of linc-ROR in maintaining the pancreatic CSLCs properities. Compliance with its role in iPS cells, we found that linc-ROR expression relates to stemness in pancreatic cancer cell. Our data showed that knockdown of linc-ROR reduced sphere formation, diminished CSC marker expression, and impaired tumorigenesis.

MicroRNAs have been implicated in the regulation of CSC properties including cell-cycle, differentiation, migration, invasion and EMT, which contribute to improve Initiation and metastasis of tumor.^38,39^ Except miR-145 which was identified as a target of linc-ROR, Hou *et al.*^15^ discovered that linc-ROR increases breast cancer cells EMT via sponge mir-205; meanwhile, their data also demonstrated that let-7a-5p mimics treatment decreased about 20 percent of Rluc activity in cells transfected with luciferase reporter gene containing linc-ROR complementary DNA (cDNA) compared with controls.^15^ Wang *et al.*^21^ also reported that miR-181a-5p and miR-99b-3p expression were enhanced in linc-ROR transcript besides miR-145-5p. The above findings suggested that linc-ROR could sponge some different microRNAs. To explore how linc-ROR function during formation of pancreatic CSLCs, we picked miRs with potential importance in inhibiting CSC properties in pancreatic CSLCs through comparing the microarray data with CSC-inhibiting miRs reported in published articles, and we further selectively up-regulated miRs following linc-ROR knockdown from these picked miRs. At last, we identified that several CSC-inhibiting miRs, including several members of let-7 family, miR-93-5p, miR-320a, miR-320b, and miR-145-3p could be enriched in linc-ROR transcript. It should be noted that the degree of enchainment of miRs were not consistent with the levels of up-regulation of these miRs mediated by linc-ROR known-down. One possible assumption for this inconsistence might be that linc-ROR could regulate microRNA expression through indirect pathway. For example, miR-320 demonstrated a inhibitory effect on Wnt/beta-catenin signaling pathway,^40^ which regulates the expression of several CSC-related microRNA such as miR-34,^41^ miR-302,^42^ and let-7.^43^ The down-regulation of other miRs might be caused by other cellular process mediated by linc-ROR via sponge several key microRNAs.

To summarize, the present work identifies linc-ROR as a novel potential oncogene in pancreatic cancer through contributing to PDAC proliferation, dedifferentiation, and stemness. We also identified some potential target microRNAs of linc-ROR in maintaining the CSC properties of pancreatic CSLCs. Owing to the vital role of these features in PDAC, we propose linc-ROR as a promising target of new therapies for this this intractable malignancy.

## Materials and methods

### Cell culture

The human pancreatic cancer cell lines PANC-1 and SW1990 were gained from American Type Culture Collection (Manassas, VA) and grown in DMEM (Gibco, NY, USA) supplemented with 10% FBS, 100 U/ml penicillin (100 U/ml, Sigma, St Louis, MO, USA) and 100 U/ml streptomycin (100 *μ*g/ml, Sigma), in a humidified 5% CO2 atmosphere at 37 °C. To enrich the CSC-like cells, chemotherapy sorting and suspension culture supporting proliferation of undifferentiated cells were adopted.^36,44–46^ Briefly, 1×10^6^ PANC-1 cells were given subcutaneous injection into the low right axilla nude mice. When the tumor volume reached ~250 mm^3^, the tumors were treated with 100 mg/kg gemcitabine twice weekly. Xenograft were removed after treated with gemcitabine for 4 weeks, excised, and evenly re-implanted in additional nude mice treated with 100 mg/kg gemcitabine twice weekly as well. After 4 weeks, the second passage xenograft were removed, excised, washed in Hanks three times, and shredded in serum-free DMEM added with 200 U/ml type IV collagenase (Sigma). Then the cell suspension was thrice filtered through 70 *μ*M nylon filters, followed by 800 r/min centrifugation. Cells were re-suspended in DMEM medium containing penicillin, streptomycin, 0.4% bovine serum albumin (Sigma), B-27 supplement (1 : 50; Gibco, Carlsbad, CA, USA), 20 ng/ml epidermal growth factor (Invitrogen, Carlsbad, CA) and 20 ng/ml basic fibroblast growth factor (Invitrogen) in low-adhesion culture bottle (Corning, NY, USA) at a density of 10^4^ cells/ml.

### Vector construction and virus infection

The pMKO.1-puro retroviral vector was purchased from Sigma. The scrambled control shRNA and linc-ROR shRNA were implanted into the pMKO.1-puro vector: linc-ROR, forward, 5′- CCGGAGGAGAGGAAGCCTGAGAGTCTCGAGACTCTCAGGCTTCCTCTCCTTTTTTG-3′ and reverse, 5′- AATTCAAAAAAGGAGAGGAAGCCTGAGAGTCTCGAGACTCTCAGGCTTCCTCTCCT-3′; scrambled control shRNA, forward 5′- CCGGTTTCTCCGAACGTGTCACGTCTCGAGACGTGACACGTTCGGAGAATTTTTG-3′and reverse,5′- AATTCAAAAAGTTCTCCGAACGTGTCACGTCTCGAGACGTGACACGTTCGGAGAA-3′. The complementary DNA of linc-ROR was purchased from RiboBio Biological Company (Guangzhou, China), and was implanted into the *Eco*R1 site of pcDNA3 (+) vector. packaging virus, infecting cells and selecting puromycin-resistant cells were described in previous research.^47^

### RNA isolation and quantitative real-time PCR

Total RNA of pancreatic cancer tissue or cells was extracted, then was converted to cDNA were performed, as previously described.^48^ Quantitative real time PCR for quantification of linc-ROR, *β*-actin (as internal control) and other mRNA was executed on a Roche Light-Cycler system (Roche, Basel, Switzerland) by using SYBR Green reaction mix (Qiagen, Foster City, CA, USA) (PCR primers sequences are supplied in Supplementary Materials). Quantitative miRNA levels were determined using real-time RT-PCR with the Applied Biosystems 7900 HT Sequence Detection System (Thermo Fisher Scientific, Waltham, MA, USA), TaqManH Gene Expression Assay (Thermo Fisher Scientific) for human let-7c-3p (assay ID 002479), hsa-let-7f-1-3p (assay ID 002417), hsa-let-7a-3p (assay ID 002307), hsa-let-7i-5p (assay ID 002221), hsa-let-7f-2-3p (assay ID 002418), hsa-let-7b-3p (assay ID 002404), hsa-let-7g-5p (assay ID 002282), hsa-let-7e-3p (assay ID 002407), hsa-let-7i-3p (assay ID 002172), hsa-let-7b-5p (assay ID 002619), hsa-let-7g-3p (assay ID 002118), hsa-let-7c-5p (assay ID 000379), hsa-let-7e-5p (assay ID 002406), hsa-let-7d-5p (assay ID 002283), hsa-let-7f-5p (assay ID 000382), hsa-let-7d-3p (assay ID 001178), hsa-let-7a-5p (assay ID 000377), hsa-miR-320a (assay ID 002277), hsa-miR-320b (assay ID 002844), has-miR-7-5p (assay ID 000268), has-miR-7-1-3p (assay ID 001338), has-miR-93-5p (assay ID 001090), has-miR-93-3p (assay ID 002139), has-miR-128-3p (assay ID 002216), has-miR-451a (assay ID 001105), and U6 snRNA (assay ID 001973) as an endogenous control. Reverse transcriptase reactions were performed by 10 ng of total RNA wich TaqMan Universal PCR Master Mix No AmpErase (Thermo Fisher Scientific) and respective TaqManH reagents. Real-time PCR was done in a total volume of 20 *μ*l reaction mixture according to the manufacture’s protocol. The reactions were incubated in 96-well optical plates at 95 °C for 10 min, and then followed by 40 cycles of 95 °C for 15 s and 60 °C for 1 min. Relative gene expression levels of mRNA and miRNAs were determined using the ^ΔΔ^Ct-method.

### Immunoblotting

Immunoblotting were executed as described previously.^47^ Simply, cells were washed with PBS and lysed in RIPA buffer (Invitrogen) added protease inhibitor (Sigma). Protein concentration of each sample was calculate by BCA protein assay kit (Thermo Fisher Scientific) to equal protein loading. Equivalent protein were underwent to SDS-PAGE, shifted to polyvinylidene fluoride membrane, interdicted in 5% skim milk for about 2 h at about 25 °C, then checked with relative primary antibodies. The following antibodies were used for analysis: anti-E-cadherin, anti-N-cadherin, anti-Vimentin and anti-SOX2 (Abcam plc, Cambridge, UK), anti-Nanog (Cell Signaling, Beverly, MA, USA), anti-*β*-actin (Sigma) and anti-*β*-tubulin (BD Biosciences, CA, USA). *β*-actin and *β*-tubulin was served as loading controls. Horseradish Peroxidase (HRP) Secondary Antibodies (Abcam) and an ECL Chemiluminescence Detection Kit (Thermo Fisher Scientific) were used to test combined antibody.

### Cell proliferation analysis

Cell Proliferation Kit I (MTT) (Sigma) was used to test cell proliferation consistent with the manufacturer’s instructions. Concisely, cells were adhered and grown in 96-well dishes with a density of 5×10^3^ cells/well overnight. The proliferation curves of cells, involving cultured cells in three days, were formulated by measuring absorbance at 570 nm.

### Flow cytometry analysis

Single cell suspensions with PBS were washed twice and 2×10^6^ cells/tube were incubated cell cycle detection reagent or fluorescent-labeled monoclonal antibodies at 4 °C in dark for 30 min. For cell cycle analysis, propidium iodide (PI, Molecular Probes, BD Biosciences) was used in staining. For detecting CSC markers, the ALDH1-PE and CD133-APC (BD Biosciences) antibodies were used. Cells were washed twice with 2 ml PBS, and then re-suspended in 200 ml PBS, last analyzed using Flow Cytometer (FACSVerse, BD Biosciences).

### Migration and invasion assays

For cell migration assays, pancreatic cancer cells were seeded into 6-well culture plates by 2×10^5^ cells/well. After 48 h, the attached cells were lightly scarred using a sterile 10-*μ*l tip and was cultured for an additional 12 h by serum-free medium. Invasion experiments were implemented by using 8-*μ*m pore size Transwell plates (Corning). Briefly, 5×10^4^ cells resuspended in serum-free medium was added to the upper wells. For promoting migration or invasion, complete medium was added to the under wells. After cultivation for 24–48 h, cells, non-penetrating through the membrane, were wiped by a swab, and the cells that were adhered to the bottom surfaces of the membrane were dyed using 0.1% crystal violet for 20 min. The number of cells adhered to the bottom surface was counted under the microscope.

### Transfection of siRNA

siRNA targeting linc-ROR were selected and transfected as described by Takahashi *et al.*^14^ siRNA against linc-ROR: 5′- GGAGAGGAAGCCTGAGAGT-3′; non-targeting control (siC) siRNA were purchased from Dharmacon (Lafayette, CO, USA). miRNA were obtained from RiboBio (Guangzhou, China). Transfections were completed with Lipofectamine 2000 (Invitrogen) for 48 h before further experiments.

### RNA-binding protein immunoprecipitation (RIP) assay

The MS2bp-MS2bs-based RIP assay was achieved consistent with previous published paper^13^ by the EZ-Magna RIP™ Kit (Merck Millipore, Munich, Germany). Briefly, cells of spheres (5×10^6^) were seeded into 100 mm plate and incubated overnight then co-transfected with 20 *μ*g linc-ROR-MS2bs overexpressing vectors (pcDNA3-ROR-MS2bs) or blank control vectors with Renilla luciferase inserts (pcDNA3-MS2bs-Rluc), 5 *μ*g FLAG-MS2bp overexpressing plasmid and indicated microRNA minics molecules (100 nM for each minics) with Lipofectamine 2000. After 48 h, 1×10^7^ cells were collected and lysed with RIP lysis buffer supplemented with 80 U/ml ribonuclease Inhibitor (RNasin, Promega, Madison, WI, USA). ANTI-FLAG M2 Magnetic Beads were used for isolation FLAG-MS2bp-MS2bs-linc-ROR complex. To evaluating the enrichment of miRNAs in linc-ROR, the ANTI-FLAG M2 Affinity Gel (Sigma) was used in RIP assay. The complexes of RNA and RNA-binding proteins were then treated with Trizol (Invitrogen), RNA was extracted and further detected by PCR.

### Sphere-formation assays

Single cell suspensions (5×10^3^ cells/ml) were seeded into low adhesion six-well plates (Corning) and incubated in modified DMEM as described in ‘Cell culture’ section without supplementation of serum. Media was replaced every 3 days. Spheres were counted after 14 days (passage one, P1). The number of secondary spheres (passage two, P2) formed following 10-days incubation after scattering was counted.

### Patients and clinical specimens

Fresh frozen tumor and para-tumor tissues were gained during resection of pancreatic cancer in Sun Yat-sen Memorial Hospital, The tissues were quick-frozen in liquid-nitrogen and deposited at −80 °C until tested. Formalin-fixed and paraffin-embedded tissues were obtained from the pathology department. The patients should sign informed consent before sample collection. Furthermore the committees of hospital should approve ethical review of the research. The patients had not received chemo- or radio-therapy before operation. The histological of each primary sample was estimated through pathological review, only invasive ductal adenocarcinomas were included, and frozen samples with <70% tumors cellularity were excluded.

### *In vivo* tumorigenesis assay

Five weeks-old male BALB/c nude mice were achieved from the Laboratory Animal Center, Zhongshan Medical School of Sun Yat-sen University and raised in laminar flow cabinets under specific pathogen-free (SPF) conditions. All procedures of experiment linking to animals were consistent with the Guide for the Care and Use of Laboratory Animals and were implemented along with the institutional ethical guidelines for animal experiment. The study protocol was also approved by the Committee on the Use of Live Animals in Teaching and Research, Sun Yat-sen Memorial Hospital, Sun Yat-sen University.

PANC-1 cells and PANC-1-derived sphere (shperes of 100–200 *μ*m in diameter were selected) were collected, dissociated to single cell by trypsin-EDTA, washed in PBS, counted and injected into the low right axilla of each mice. Tumor size was measured with the following formula: volume=(*L*×*W*^2^)/2, where *L* and *W* are the longest and shortest diameters, respectively. Mice were killed when the average L of any group reached about 1 cm.
